# Study on bacteriostasis of Chinese herbal medicine extracts to avibacterium paragallinarum

**DOI:** 10.1016/j.psj.2026.106474

**Published:** 2026-01-18

**Authors:** Zipeng Yue, Yurong Li, Ligong Chen, Tian Liang, Yinghui He, Linyao Hao, Ying Jiang, Tianyang Li, Shuying Huo

**Affiliations:** a College of Veterinary Medicine, Hebei Agricultural University, Baoding 071000, China; b College of Life Sciences, Hebei University, Baoding 071000, China

**Keywords:** Chinese herbal medicine, Avibacterium paragallinarum, Bacteriostatic effect, MIC_80_

## Abstract

To explore the treatment of Chinese herbal medicine extracts on infectious rhinitis caused by Avibacterium paragallinarum, a strain of Avibacterium paragallinarum was isolated from the nasal cavity and infraorbital sinus of chickens suspected of infectious rhinitis, it was biologically identified as *Bacillus paracarinus*. Extracts of seven kinds of traditional Chinese medicine and traditional Chinese medicine mixtures were explored to determine the minimum inhibitory concentration (MIC) by the microdilution method and the disc diffusion method. The antibacterial activity against pathogenic bacteria of Chinese herbal medicine extracts was determined by the animal infection treatment test. The MIC_80_ results showed that the Acorus gramineus, Phellodendron chinense and Herba taraxaci were 4 μg/μL, the Sophora flavescens was 16 μg/μL, the Pulsatilla adans was 32 μg/μL, and the Cassia obtusifolia and Polygonum hydropiper were 128 μg/μL, while the minimum MIC_80_ of Mixture Ⅰ was 1 μg/μL. The results of the drug susceptibility test of the paper diffusion test, while the inhibition zone of Mixture Ⅰ was the largest, with an inhibition zone of 2.16 ±0.15. It shows that the mixture Ⅰ had a strong inhibitory effect on Avibacterium paragallinarum. The results of animal experiments showed that the average daily gain of chicks in the protection group and the treatment group was higher than that in the infection group. A qPCR assay was performed to verify that mixture I could reduce the expression of inflammatory factors. Conclusion: 7 kinds of Chinese herbal medicines, including calamus, dandelion, and phellodendron, exhibited better antibacterial effects against Avibacterium paragallinarum. Additionally, Mixture I showed significant preventive and therapeutic effects on infectious coryza in chickens caused by Avibacterium paragallinarum. Furthermore, Mixture I could effectively reduce the expression of inflammatory factors in macrophages that were stimulated with lipopolysaccharides.

## Introduction

Respiratory diseases have been a significant cause of economic losses in the poultry industry. At the same time, there has been a growing need to prevent the emergence of antibiotic resistance and the presence of antibiotic residues in widely consumed meat products.

Avian infectious coryza, a highly contagious respiratory disease caused by Avibacterium paragallinarum (formerly known as Bacillus paracarinus), has become increasingly severe in recent years and has been reported worldwide (Rajeswar et al., 2022). This acute airborne disease was characterized by rapid transmission, typically spreading to the entire flock within three to five days. Infected chickens exhibited a high incidence of clinical signs, including sneezing, frequent head shaking, conjunctivitis, and periorbital swelling. In severe cases, blindness was observed (Yang et al., 2019). Chickens infected during the growing phase showed stunted growth, while laying hens experienced a significant decline in egg production (Wahyuni et al., 2018).

The disease has had a global impact, often exacerbated by co-infections with respiratory pathogens such as Mycoplasma synoviae, Mycoplasma gallisepticum, Pasteurella spp., and infectious bronchitis virus, as well as various stress factors (Akter et al., 2013). Economic losses resulted from reduced egg production (10 %-40 %) and increased culling rates during the growth period (Sun et al., 2018).

In recent years, long-term misuse of antibiotics in livestock farming has led to increased bacterial resistance. In response, China has fully implemented policies to prohibit, reduce, and restrict antibiotic use in animal production. Consequently, no suitable conventional drugs remained available for treating infectious coryza. This situation has shifted research attention to Chinese herbal medicines as natural alternatives, particularly focusing on active ingredients extracted from medicinal plants (Hong et al., 2018; Dong et al., 2014).

Chinese herbal medicines were considered promising due to their low toxicity, minimal side effects, low tendency to induce resistance, and synergistic effects of multiple active components (Yang et al., 2021). Consequently, the search for safe, natural antibacterial agents became an important research trend aimed at protecting animal health and ensuring human safety.

Several herbs, including Cassia seed (Cassia obtusifolia), Acorus gramineus, Phellodendron chinense, Polygonum hydropiper, Sophora flavescens, dandelion (Herba taraxaci), and Pulsatilla chinensis, were reported to possess pharmacological properties such as anti-inflammatory, antiparasitic, heat-clearing, dampness-drying, and antibacterial activities (Deshpande and Naik, 2016; Helaly et al., 2019; Rui et al., 2013; Zhang et al., 2021).

Therefore, this study aimed to investigate the antibacterial activity of these seven Chinese herbs against Avibacterium paragallinarum and to identify potential natural agents for the treatment of infectious coryza. Since the official implementation of antibiotic restriction policies in China’s aquaculture industry in 2020, the incidence of such diseases has been rising year by year (Wu et al., 2021).

## Materials and methods

This study was approved by the Experimental Animal Ethics Committee of Hebei Agricultural University. Official approval number:2023087.

### Sick material

Sick materials were collected from sick chickens delivered to Nongxiang Livestock and Poultry Clinic in Baoding City, Hebei Province. The sick chickens showed unhealthy, with fluffy feathers, swelling of the head and face, large amounts of mucus in the infraorbital sinuses and nasal cavity, and jelly-like ooze under the skin of the head. Insert a sterile swab deep into the infraorbital sinus or nasal cavity and rotate to collect mucus and exudate. Then, Place the cotton swab with the mucus and exudate into 10 mL of sterile normal saline for dilution. To prevent horizontal bacterial infection, each group of experimental animals was separately raised in a sterile environment. Decontamination Procedures: All decontamination operations must be conducted within their respective compartments. Operational Sequence: Daily operations must follow the principle of “clean to contaminated.” Sampling must be completed within each compartment. Before removing sample tubes from the compartment, their exteriors must be thoroughly wiped with disinfectant and placed into sealed secondary containers.

### Test animals

20 five-week-old white-feathered broilers, and 100 two-week-old white leghorn chickens were provided by Dingnong Group, Baoding City, Hebei Province, China.

### Main reagents

Tryptone, yeast powder, sodium chloride, agar, and nutrient agar were purchased from Huangzhong Harvest Biotechnology Company; Gram stain was purchased from Beijing Reagan Biotechnology Co., Ltd; goat blood was purchased from Beijing Dingguo Changsheng Biotechnology Co., Ltd; Erythromycin blue medium was purchased from Baobao Bioengineering Co., Ltd; and soybean casein agar medium Tryptone Soy Agar TSA medium was purchased from Shanghai Jizhi Biochemical Technology Co., Ltd; The primers were designed and synthesized by Shanghai Paysono Biotechnology Co., Ltd, and Acorus calamus, Cyperus rotundus, Cassia sinensis, Radix et Rhizoma Ginseng, Rhizoma Polygoni Multiflori, Rhizoma Polygoni Multiflori, and Radix et Rhizoma Dandelion were purchased from Anguo Herbal Market. Inflammatory factor primers, RNA extraction kit (G3640), reverse transcription kit (G3333), and SYBR Green QpcrMaster MiX (G3326) were purchased from Wuhan Xavier Biotechnology Co., Ltd; MTT powder and Neutral Red dye were purchased from Solepol.

### Main instruments

Incubator, purchased from Shanghai Formosa Experimental Equipment Co., Ltd; ultrasonic extractor, purchased from Shanghai Yumo Instrument Co., Ltd; SHZ-D Circulating Water Multipurpose Vacuum Pump (purchased from Henan Yuhua Instrument Co., Ltd., China), and Real-Time Fluorescence Quantitative PCR Instrument (Model: Gentier 48R; Manufacturer: Tianlong Technology, China).

## Methods

### Extraction of Chinese herbal medicine

Twenty grams of pulsatilla chinensis, cassia seed,acorus gramineus, phellodendr on chinense, polygonum hydropiper, sophora flavescens, and dandelion were soaked overnight in a 60 % ethanol. The extraction of Chinese herbs was adapted and improved from the ultrasonic-alcohol extraction method described by (Hui et al., 2022; Zhang et al., 2020). The herbs were soaked in 60 % ethanol solution at a 1:10 (w/v) herb-to-solvent ratio, incubated at room temperature for 24 h, and then transferred to an ultrasonic extractor. Ultrasonic extraction was performed at 45 °C for 15 min, and this extraction process was repeated three times. Subsequently, the residue was transferred to a vacuum filter flask and filtered under vacuum twice. After extraction and filtration, the residue was re-soaked in 60 % ethanol at a 1:6 (w/v) ratio, and ultrasonic extraction and filtration were repeated as described above. The resulting liquid extracts were combined and centrifuged at 3000 r/min for 10 min. After centrifugation, the supernatant of each sample was transferred to a rotary evaporator at 80 °C, where the extracts of the seven Chinese herbs were concentrated to a final concentration of 2 g/mL and stored at 4 °C in the dark for subsequent use. Traditional Chinese medicine mixture I was prepared by combining Acorus tatarinowii (calamus) and Phellodendron chinense in a 1:1 ratio; mixture II was prepared with Acorus tatarinowii and dandelion in a 1:1 ratio; mixture III was prepared with Acorus tatarinowii and dandelion in a 1:1 ratio; and mixture IV was prepared with Acorus tatarinowii, Phellodendron chinense, and dandelion in a 1:1:1 ratio. The same ingredients in different preparations are used to identify the optimal combination in order to achieve the maximum therapeutic effect. The extraction method for these mixtures was the same as described above (Zhang et al., 2020).

### Isolation and purification of pathogenic bacteria and gram staining

In a sterile environment, a cotton swab was used to collect inflammatory exudate from the nasal mucosa of chickens, and the collected exudate was then mixed with 10 mL of sterile saline for dilution. A 100 μL aliquot of the dilution was aseptically absorbed, evenly applied onto TSA medium, and incubated at 37 °C for 24 h for colony growth observation. A single colony was selected, placed on a slide, and microscopically examined under a light microscope. The streaking method was employed to isolate and purify the single colony. A purified colony of the pathogenic bacteria was uniformly smeared on a slide for Gram staining, and the bacterial morphology was observed under an oil immersion lens at 100 × magnification.

### Observation of satellite growth of pathogenic bacteria

Absorb 50 μL bacterial solution and evenly smear it on the blood AGAR plate, then dip a small amount of Staphylococcus aureus into the bacterial ring and draw a straight line at the center of the plate, and culture it at 37 °Cfor 18 h∼ 20 h to observe whether the satellite phenomenon of pathogenic bacteria growth occurs.

### Physiological and biochemical characteristics of pathogenic bacteria

The purified bacterial solution was inoculated with catalase, ornithine decarboxylase, galactose, sorbide, hydrogen sulfide and glucose assimilation tubes, and cultured at 37 °C for 24 h to observe the growth.

### Molecular biological identification

Pathogen DNA was extracted according to the bacterial DNA extraction kit, and PCR amplification was carried out with 27F5-AgagTTTGATCCTGGCTCAG 3 and 1492R5 Yones. The expected amplified fragment is about 1450 bp. PCR reaction system: 2 × Taq PCR MasterMix 25 µL, template DNA(526 ng/µL) 3 µL, upstream and downstream primers (25 µmol/µL) 1 µL each, ddH_2_O 20 µL supplemented to 50 µL. PCR amplification conditions: Pre-variable at 94 °C for 3 min; Denatured at 94 °C for 30 s, annealed at 52 °C for 30 s; Elongation at 72 °C for 1 min, 30 cycles; It was extended at 72 °C for 10 min and stored at 4 °C. After the PCR reaction, 3 µL PCR product was detected by 1 % agarose gel electrophoresis. It was sent to Shanghai Piceno Biotechnology Co., Ltd. for sequencing, and the sequencing results were compared with the registered gene sequences in the GenBank database. MEGA 7.0 was used to make an evolutionary tree.

### Animal regression test

20 five-week-old white feather broilers were randomly divided into two groups with 10 broilers in each group. The first group was a blank group, and the second group was an animal-infected group. 0.2 ml bacterial solution with a concentration of 10^8^ CFU/mL was injected into the suborbital sinus of the infected group. The pathological section of the lesion was made for histological observation, and bacteria were isolated and identified. The minimum inhibitory concentration (MIC_80_) and minimum inhibitory concentration (MIC) of Chinese herbs against Bacillus paracarinis were determined in accordance with the M27-E4 standard established by the Clinical and Laboratory Standards Institute (CLSI). The concentration of 2 g/mL Chinese medicine solution was diluted to 1024, 512, 256, 128, 64, 32, 16 and 8μg/μL with TSA liquid medium, and 100 μL of each was taken into 96-well plates, and 100 μL10^3^CFU/mL of bacterial suspension was taken into the Chinese medicine holes. The final concentration of each Chinese medicine hole was 512, 256, 128, 64, 32, 16, 8, and 4 μg/μL, the operation method of 75 % ethanol control group was the same as above, and positive control and negative control were set, and the OD600 value of each hole was cultured at 37 °C for 48 h, and the concentration of the drug whose OD value decreased by more than 80 % was MIC_80_. The experiment was repeated three times.

### Susceptibility test by the disk diffusion method

Filter paper with a diameter of 6mm was selected and put into a small bottle, autoclaved at 121 °C for 30 min, dried, and used (Yuan et al., 2020). 7 kinds of alcoholic extracts of traditional Chinese medicine with a concentration of 1 g/mL were put into small bottles (the amount of liquid is based on the impregnated paper), and the drug stock solution was prepared according to the different concentrations of various antibacterial Chinese medicines in the preparation, which was 200 times larger than the required preparation concentration. After soaking for 24h, the excess liquid was poured away, and the paper was dried and stored in the refrigerator at 4 °C for later use. Absorb 1mL bacterial solution and evenly apply it to the nutrient AGAR medium. 7 kinds of dried Chinese medicine sensitive tablets were pasted into the medium according to the region division, with 5 tablets per dish and 3 replicates. The samples were cultured in an inverted position in a 37°C incubator for 24 h, followed by observation to measure the diameter of the zone of inhibition (including the diameter of the sensitive tablets). The antibacterial susceptibility testing of western medicines was performed in accordance with the standards published by the National Committee for Clinical Laboratory Standards (NCCLS, 2013), and the results were categorized into three grades: sensitive (S), intermediate (I), and resistant (R). The criteria for judging antibacterial results of traditional Chinese medicines were as follows: the diameter of the antibacterial circle was 15-20 mm for sensitive, 10-14 mm for moderately sensitive, and less than 10 mm for insensitive.

### Animal infection treatment trials

A total of 100 two-week-old white leghorn chickens were randomly divided into five groups (Zhang et al., 2020). Group 1 was the blank control group, Group 2 was the experimental group with simple infection, Group 3 was the protection group given Chinese medicine before infection test, Group 4 was given sulfadiazine after infection, and Group 5 was the treatment group given Chinese medicine after infection, with 20 chickens in each group. The experimental animals were weighed daily before feeding, and the daily feeding rate was (day age +2) g. To prevent bacterial infection, the experimental animals in each group were raised separately in a sterile environment. Before the infection test, the experimental animals in the third group were orally given 1 g/mL concentration of traditional Chinese medicine mixture Ⅰ (daily feeding rate × 1.5 %) mL, and the other groups were fed normally. 7 days later, 0.2 mL of 10^8^ CFU of Paracarinella solution was injected into the suborbital sinus of chickens, and all experimental animals except group 1 were tested for infection. Until the test animals developed facial edema, sneezing, and serous or mucous secretions oozing through the nasal cavity. Finally, the experimental animals in group 5 were orally given 1g/mL of Chinese medicine mixture Ⅰ (daily feeding dose × 1.5 %), the experimental animals in group 4 were fed sulfadiazine (800 units/one), and the other groups were fed normally. 7 days later, all the test animals were dissected, the diseased organs were observed, bacteria were isolated and identified, and the initial and final weights of each group of test animals were recorded. Excluding dead chickens, the average daily gain of each group was calculated, and then the morbidity and mortality were also calculated.

### Grouping assay of raw264.7 cells

To analyze the effect of Mixture I on cellular inflammation, Raw264.7 cells were divided into control and LPS treatment groups, and corresponding DMEM was added to each well. 1 μg/mL lipopolysaccharide was used to stimulate Raw264.7 cells in the LPS group to serve as a cellular model of inflammation for the subsequent study. A group of traditional Chinese medicine (TCM) at a concentration of 10^-4^ as well as a mixed group of TCM plus lipopolysaccharide were set up (the initial concentration of 1 g/ml was diluted to 10^-4^ g/ml, and the LPS concentration was 1 μg/mL). The initial concentration of Chinese herbal medicine was 1 g/ml diluted to 10⁻⁴ g/ml, and the concentration of LPS was 1 μg/mL. The Chinese herbal medicine plus lipopolysaccharide group was defined as the addition of Chinese herbal medicine to cells cultured in lipopolysaccharide for 24 h. Adding Chinese herbal medicine to cells aimed to reduce the excessive release of inflammatory factors that would lead to inflammatory exacerbation.

### Viability and phagocytosis assay of raw264.7cell

Raw264.7 cells were divided into 6 groups: control (0), 10^-2^, 10^-3^, 10^-4^, 10^-5^, and 10^-6^ (in DMEM). 96-well cell culture plates were inoculated at a density of 1 × 105/mL with 3 replicates in each group, and the cells were allowed to grow up to 70 % or for about 24 hours before drug intervention. The cells were incubated at 37°C in a 5 % CO₂ incubator for 24 h, and then MTT solution (5 mg/mL) was added to each well and incubated for 4 h. The absorbance of the samples at 450 nm was measured using an enzyme meter to calculate the effect of DMEM on cell viability. The phagocytosis ability of the macrophage RAW264.7 was measured by the neutral red method. Macrophages in the logarithmic growth phase were harvested, and their cell density was adjusted to 1 × 10^5^ mL. The cell suspension was then seeded into 96-well cell culture plates and treated with test drugs at respective concentrations for 24 h. After drug treatment, neutral red solution was added, followed by further incubation. The absorbance at 540 nm was measured, and the phagocytic rate of macrophages was calculated using the Neutral Red Assay according to the absorbance values.

### Real-time quantitative PCR method

The total RNA of Raw264.7 cells was extracted by RNA extraction reagent, and the concentration of total RNA was determined by ultra-micro spectrophotometer, and the total RNA was transcribed into cDNA by using a reverse transcription kit, and the Ct values of interleukin 1β (IL-1β), TNF-α, and IL-6 mRNA were measured by real-time PCR, with β-actin as the internal reference gene. The Ct values of interleukin-1β (IL1β), TNF-α, and IL6 mRNAs in Raw2642.7 cells were determined by using β-actin as the internal reference gene. The PCR reaction system had a total volume of 20 μL, consisting of the following components: 2 × SYBR Green qPCR Master Mix 10 μL, 2 μL each of upstream and downstream primers, 1 μL of cDNA samples, and 7 μL of ddH₂O. The PCR reaction program was set as follows: pre-denaturation at 95°C for 120 s, followed by 40 cycles of denaturation at 95°C for 15 s and annealing at 60°C for 30 s. The relative expression of each gene was calculated by the 2^-△△^Ct method. Primer information is detailed in Table 1.

**Table 1:** Primer sequences.

Gene	Primer sequences(5′→3′)	Annealing temperature/ °C
β-actin	F:GTGACGTTGACATCCGTAAAGA R:GTAACAGTCCGCCTAGAAGCAC	60
IL1β	F: GGGCTGCTTCCAAACCTT R:GAGTGATACTGCCTGCCTGAA	59
IL6	F:AATGATGGATGCTACCAAACTG R:TTGGATGGTCTTGGTCCTTAG	61
TNF-α	F: GGTGCCTATGTCTCAGCCTCTT R:GCCATAGAACTGATGAGAGGGAG	60

### Data analysis

All the experiments were repeated at least three times, and the results were expressed as means ± SE. Statistical analyses were performed using the SPSS software package V11.5 (SPSS Inc., Chicago, IL). All data were analyzed using one-way analysis of variance (ANOVA) to determine the differences among the groups. In this study, *P* < 0.01 is an extremely significant difference, and *P* < 0.05 is a significant difference.

## Results

### Morphology of pathogen

**Fig. 1:**
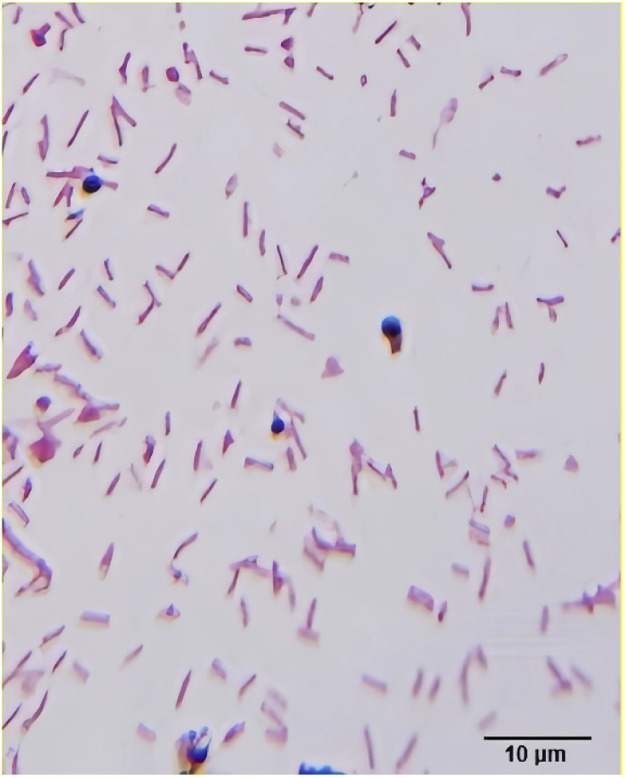
The Gram staining results were obtainedogy (Gram staining, 100 ×).

The pathogens on the TSA medium were round, smooth, grayish white, and translucent and dew-like colonies with a diameter of about 0.3mm on the TSA plate, with smooth surfaces, negative gram staining (Fig. 1). It was small and rod-shaped, which was consistent with the growth morphology of Paracarinella.

### Satellite observation

**Fig. 2:**
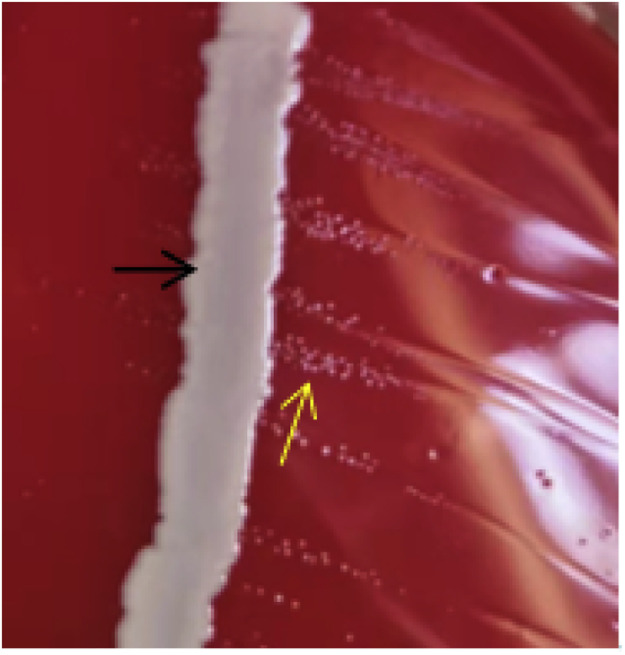
Satellite phenomenon. Black arrow: Staphylococcus aureus; yellow arrow: Avibacterium paragallinarum.

As shown in Fig. 2, a satellite phenomenon of colony growth appeared in the blood AGAR medium after 18 h of culture, and pathogens and Staphylococcus aureus were present in the medium at the same time. The pathogenic bacteria grew more densely around Staphylococcus aureus, while the pathogenic bacteria colonies in the peripheral part of Staphylococcus aureus were relatively sparse.

### Physiological and biochemical characteristics

According to the culture time specified in the instructions of the microbiochemical identification tube of bacteria, the results showed that the isolate did not grow in the medium without factor V, and the reactions of catalase, sorbitol, galactose, glucose and galactose were negative (Table 2), which was in line with the biochemical characteristics of avian Bacillus paracarinus.

**Table 2:** Results of the physiological and biochemical tests.

project	Catalase	Ornithine decarboxylase	Galactose	Grow in atmosphere	Does the growth environment require factor V	Sorbitol	Hydrogen sulfide	Glucose
result	-	-	-	-	+	-	-	-

### Results of molecular biological identification

**Fig. 3:**
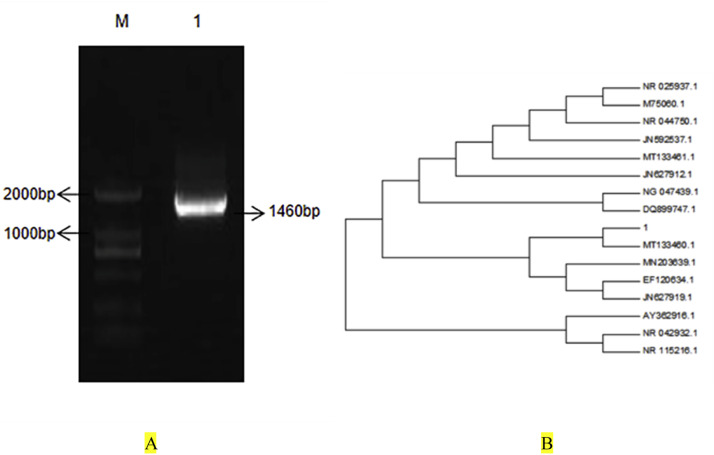
PCR detection results and phylogenetic tree of the pathogen 16SrDNA A, PCR detection results of pathogen 16SrDNA; B, target bacterial phylogenetic tree.

PCR results of 16S rDNA of the pathogen showed that the PCR amplification of the pathogen produced a 1460 bp band (Fig. 3-a). BLAST comparison revealed that the sequence homology of the pathogen and P. paraculatus was 99.9 %. Phylogenetic tree analysis showed that the pathogen was most closely related to strain MT133460.1 of P. Paraculatus. The pathogenic bacterium was identified as a strain of Paracarinus and named as 1 (Fig. 3-b).

### Results of pathogenic regression test

**Fig. 4:**
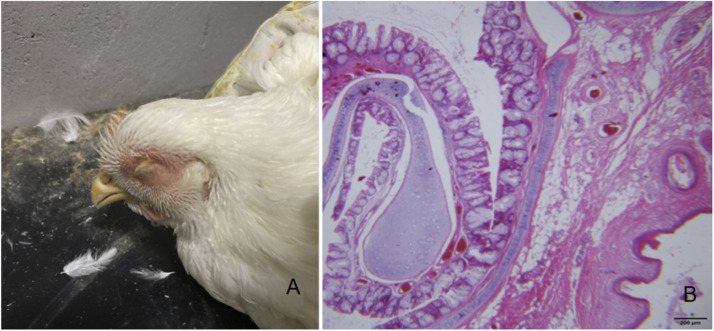
Clinical symptoms and histopathological observation (H.E. stain 40 ×) A, Clinical symptoms; B, Histopathological observation.

After 24 hours of inoculation, the chickens in the experimental group showed facial swelling, low energy, head shaking, and closed eyes (Fig. 4-a). Subcutaneous peptone-like exudation of the infraorbital sinus was found after autopsy. Meanwhile, the lesions of the respiratory system were mainly located in the pharynx and larynx of the upper respiratory tract, and there was a large amount of mucus in the lumen. Besides, no significant pathological changes were observed in their heart, livers, spleens, kidneys, and other tissues. H.E. staining results of pathological sections showed that there was a large amount of mucous material in the nasal cavity and inflammatory cell infiltration accompanied by bleeding (Fig. 4-b). The bacteria isolated from the lesion site were identified by biochemical and molecular biology, and were identified as the bacteria for this experiment.

### Results of MIC_80_ test

As shown in Table 3, the determination results of MIC_80_ of paracarinella were as follows: the bacteriostatic rate ≥80 % when the concentration of mixtureⅠ was 1μg/μL, the bacteriostatic rate ≥80 % when the concentration of Phellodendrin, Gladiolus and dandelion was 4 μg/μL, the bacteriostatic rate ≥80 % when the concentration of sophora was 16 μg/μL, and the bacteriostatic rate ≥80 % when the concentration of pulsatila was 32 μg/μL. Cassia seed and spicy manifold had no significant inhibitory effect on its growth.

**Table 3:** MIC_80_ measurement results.

MIC_80_ (μg/μL)	Phellodendron chinensis	Acorus gramineus	Herba taraxaci	Sophora flavescens	Pulsatilla adans	Cassia obtusifolia	Polygonum hydropiper
Avibacterium paragallinarum	4	4	4	16	32	128	128

MIC_80_ (μg/μL)	MixtureⅠ		Mixture Ⅱ		MixtureⅢ	MixtureⅣ	
Avibacterium paragallinarum	1		64		16	64	

Note: MIC_80_ means comparing with the negative control, the lowest drug concentration with an OD_630_ value declining more than 80 %.Abbreviation: MIC, minimum inhibitory concentration

### Paper diffusion method for drug sensitivity test

The results of the drug sensitivity test are shown in Table 4. The avibacterium paragallinarum was sensitive to mixture Ⅰ ,mixtureⅡ, mixture Ⅲ, mixtureⅣ, Phellodendron chinense, Herba taraxa, and Cassia obtusifolia. While it displayed intermediate susceptibility to Pulsatilla adans, Acorus gramineus, Sophora flavescens, and Polygonum hydropiper.

**Table 4:** Disk diffusion susceptibility testing.

Chinese herbal medicine name	Gentamicin	Phellodendron chinense	Herba taraxaci	Cassia obtusifolia	Pulsatilla adans	Acorus gramineus	Sophora flavescens	Polygonum hydropiper
Zone of Inhibition Diameter(cm)	2.23±0.05	1.91±0.03	1.80	1.50	1.41±0.03	1.40	1.03±0.03	0.98±0.03
Degree of Sensitivity	S	S	S	I	I	I	I	I

Chinese herbal medicine name	mixture Ⅰ		mixtureⅡ		mixture Ⅲ		mixtureⅣ	
Zone of Inhibition Diameter(cm)	2.17±0.15a		1.73±0.06b		1.87±0.06b		1.80±0.01b	
Degree of Sensitivity	S		S		S		S	

Note: Within a column, values with different lowercase superscript letters differ significantly (*P* < 0.05), while those with the same letter do not differ significantly (*P* > 0.05). According to the inhibition zone diameter, susceptible (S) is defined as 15 mm–20 mm, intermediate (I) as 10 mm–14 mm, and resistant (R) as < 10 mm.

### Experimental results of animal infection treatment

As shown in Table 5, the average daily weight gain of the first group was higher than that of the other groups, followed by the higher average daily weight gain of the third and fifth groups, and the lowest average daily weight gain of the group 2. The mortality rate in the first group was 0 %. The incidence rate of Groups 3, 4 and 5 was lower than that of Group 2. After infection with Haemophilus parahaemolyticus, the growth of laying hens in groups 3 and 5 stagnated during the experiment. The group 2 of laying hens grew the slowest. Compared with the prevention group and the treatment group of Chinese herbal mixture I, the average daily feed intake of the infected group was significantly lower *P*<0.05. The material weight ratio in the western medicine treatment group was significantly increased *P*<0.05. It could be seen that Mixture Ⅰ had protective and therapeutic effects on chickens infected with Haemophilus parahaemolyticus.

**Table 5:** Mortality and average daily gain in animal experiments.

Group	1	2	3	4	5
Average starting weight (g)	75.72	67.06	63.53	74.38	69.09
Average final weight (g)	194.0	140.33	165.31	148.75	168.0
Average daily intake	22.98±0.94^a^	16.64±0.56^d^	20.66±0.71^b^	17.92±0.26^c^	20.5±0.51^b^
Average daily gain (g)	6.95±0.387^a^	4.31±0.452^b^	5.99±0.389^a^	4.37±0.33^b^	5.82±0.109^a^
Material weight ratio	3.55±0.17^ab^	3.77±0.42^ab^	3.46±0.19^b^	4.32±0.45^a^	3.52±0.28^ab^
Incidence( %)	0	53.84	15.38	25	9.09
Mortality rate ( %)	0	13.33	0	0	0

Note: Different lowercase letters in the same column indicate significant differences (*P* < 0.05), while the same lowercase letters indicate no significant differences (*P* > 0.05).

### Raw264.7cell activity and phagocytosis assay

**Fig. 5:**
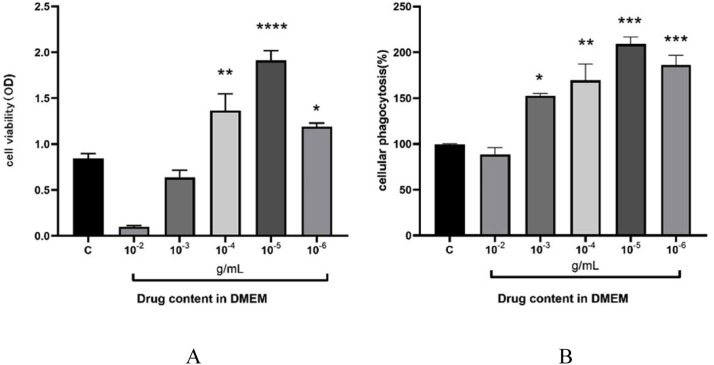
A shows cell viability, and Fig. 5-B shows cell phagocytic ability.

The cell activity assay was mainly tested by MTT. The initial concentration of the drug was 1 g/mL, and at a concentration of 10^-5^ g/mL, the cell viability was the strongest as the optimum concentration for the study (Fig. 5-A). The phagocytosis assay was performed using a neutral red dye, and finally, the strongest phagocytosis was achieved at a concentration of 10^-5^ g/mL (Fig. 5-B).

### Effect of the combination of phellodendron bark and acorus calamus on cellular inflammatory factors

**Fig. 6:**
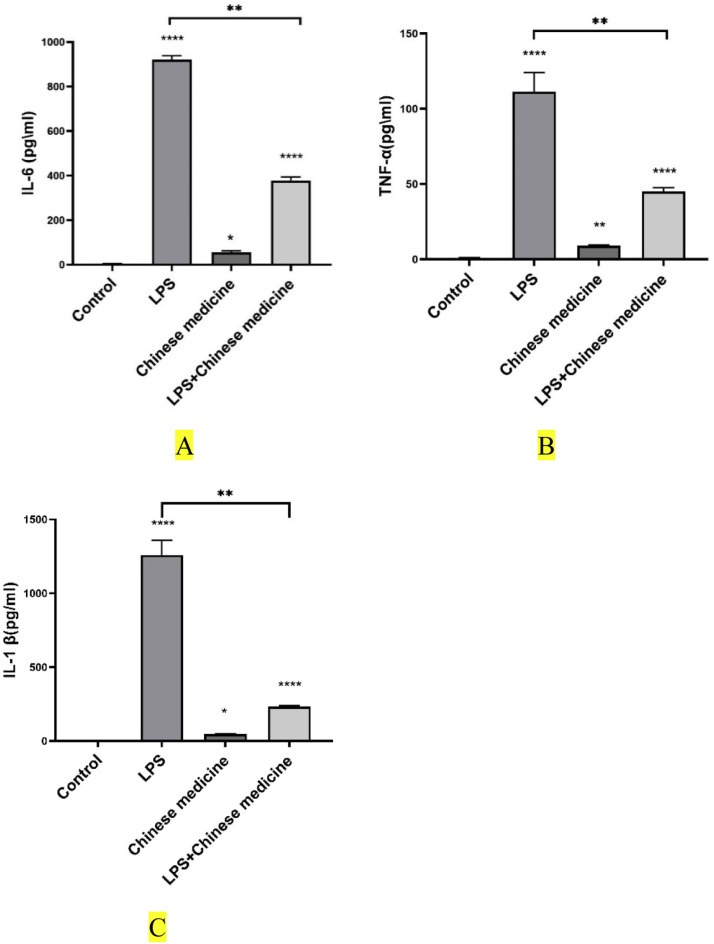
The effect of a compound preparation of Phellodendron and Acorus calamus on cellular inflammatory factors. Note: *P < 0.05, **P < 0.01, **P < 0.001*.*

As can be seen in Fig. 6, compared with the control group, the expression levels of IL-6(6-A), IL-1β(6-B), and TNF-α(6-C) in cells of the traditional Chinese medicine group were increased; compared with the model group, the expression levels of IL-6, IL-1β, and TNF-α in cells of the conventional Chinese medicine group were decreased, indicating that the traditional Chinese medicine group could inhibit cellular inflammatory response. The expression levels of IL-6, IL-1β, and TNF-α in the group treated with conventional Chinese herbal medicine combined with lipopolysaccharide (LPS) were lower than those in the LPS-only group.

## Discussion

It has been reported that P. paracarinis is resistant to cephalexin, amoxicillin, tetracycline, penicillin G, ceftazidime, chloramphenicol, kanamycin, cotrimoxazole, ampicillin and other drugs, so it has become particularly important to find those antibiotics derived from natural plants (Jeong et al., 2019). In this study, a gram-negative bacterium was isolated from the nasal cavity and infraorbital sinus of clinically ill chickens. The study revealed that the form of Paracarinella was relatively regular, appearing as a small and gram-negative bacillus, with dimensions of approximately (1.0-3.0) μm × (0.4-0.8) μm, without flagellum and spore formation (Rong et al., 2011). The results showed that the pathogenic bacteria were consistent with the morphological characteristics of P. paracularis. The homology of the 16SrDNA sequence was 99.9 %, and it was identified as P. paracularis. To confirm and manage the disease, isolation and accurate identification of Av. paragallinarum are essential (Kang et al., 2023). A variety of diagnostic methods, including bacteriological tests, can be used to isolate and identify Av. paragallinarum. Nonetheless, due to numerous factors that may impede the successful culturing of Av. para gallinarum, polymerase chain reaction (PCR) serves as a reliable diagnostic alternative (Putra FN et al. 2023).

Avian infectious rhinitis is a kind of upper respiratory tract disease caused by Bacillus paracarinus, which damages the nose and respiratory tract epithelium and causes edema of the face (Lin et al., 2018). In this study, the isolated Paracarinella was injected into the infraorbital sinus for an animal infection regression test. The experimental animals showed clinical symptoms, including serous or mucous nasal exudation, infraorbital sinus swelling, facial edema, and conjunctivitis 24 h after inoculation, and the pathological section was H.E. The staining of nasal inflammatory cells infiltrated and a large amount of mucus was consistent with the symptoms of infectious rhinitis, which fully proved the strong pathogenicity and infectivity of the bacterium. After a cluster outbreak of infectious rhinitis in chickens, recovered chickens carry the bacteria for a long time, and after increased exposure, there is the possibility of further repeated outbreaks of disease. A variety of factors are related to the pathogenicity of P. paracarinis, and three virulence-related antigens are mainly considered (Blackall, 1999; Ying et al., 2016), which cause great harm to the chicken industry. In this experiment, 7 kinds of Chinese herbs with antibacterial function were selected, which were dandelion, Phellodendron, calamus, cassia seed, Pulsatilla, and sophora flavescens. The ultrasonic alcohol extraction method was used to extract the effective components of traditional Chinese medicine, and the antibacterial effect of 7 kinds of traditional Chinese medicine was determined through in vitro antibacterial test. The most sensitive and effective traditional Chinese medicine mixture was selected successfully. The results revealed that when MIC_80_ was 4 μg/μL, Phellodendri, Gladiolus, and dandelion had the strongest antibacterial activity. Mixture I, with the strongest antibacterial activity, was obtained by the Chinese medicine compatibility test, and the concentration was 1 μg/μL, which could inhibit more than 80 % of paracarinella. Studies have reported that Phellodendron phellodendron contains alkaloids, flavonoids, sterols and terpenes, and other chemical components. It has pharmacological effects such as antibacterial and anti-inflammatory, lowering blood pressure and blood glucose, anticancer, immunosuppressive and anti-ulceration (Ru et al., 2020). Berberine in Phellodendron phellodensis can inhibit gram-negative and gram-positive bacteria, and has a broad-spectrum antibacterial effect. When the concentration of berberine is high, it can also achieve a bactericidal effect (Rong et al., 2016). Several studies have indicated that berberine may be effective for inflammation. The abundant pharmacological effects of Acorus acorus have made it a research focus in recent years. Relevant studies have shown that Acorus has anti-inflammatory, analgesic and neuroprotective effects (Rui et al., 2013). Previous studies investigated the antibacterial effect of Acorus acorus, and the experimental results showed that Acorus acorus exhibited antibacterial effects against Escherichia coli, Staphylococcus aureus, yeast and Shigella dysentery (Feng et al., 2016; Zhu et al., 2022). Another study found that the volatile oil of Gladiolus acorus was the main effective extraction part of Gladiolus acorus, which had anti-inflammatory and analgesic effects. The better antibacterial effect of Phellodendron phellodendron and dandelion among the 7 Chinese herbs may be related to the alkaloids, volatile oil, and α, β-asaryl ethers contained in them. The enhanced antibacterial effect of mixture Ⅰ of Phellodendron Phellodendron and Acorus calorius may be due to the synergistic effect of berberine and volatile oil active components, and the medicinal mechanism needs to be further studied.

The animal infection treatment experiment showed that the body weight and average daily gain of the experimental animals in the Chinese medicine treatment group were significantly increased, the incidence was significantly reduced, and the white blood cell count was significantly lower than that in the untreated group. White blood cells can phagocytize bacteria and play an important role in resisting the invasion of pathogens and immunity to diseases (Smith, 2009). The results of this experiment showed that mixture I had obvious protective and therapeutic effects on paracarinus infection, and the mechanism of action might be that traditional Chinese medicine increased the immune function of poultry itself, enhanced the phagocytosis function of macrophages, or regulated the specific immune response of lymphocytes, and enhanced mucosal immune function. The primary reason is that the active ingredient of Chinese medicine itself has an inhibitory effect on the growth of Bacillus paracarinus, as confirmed by our in vitro antibacterial tests. In vitro experiments have demonstrated that macrophages can secrete various cytokines, such as IL-1β, IL-6, and TNF-α, which are involved in the immune response, promotion of cell growth, and repair of damaged tissues. Meanwhile, the release of cytokines is one of the hallmarks of macrophage activation. Studies have shown that IL-1β contains a Toll-IL-1 receptor structural domain and can respond to microbial and viral stimuli. In contrast, the secretions of IL-1β, IL-6, and TNF-α were increased when LPS acted on macrophages. However, mixture I inhibited the secretion of IL-1β, TNF-α, and IL-6 by macrophages stimulated with LPS.

In summary, a strain of Paracarinella was successfully isolated from sick chickens in this experiment, and the strongest antibacterial effect of the Chinese medicine mixture I was screened out through a drug sensitivity test. The treatment effect was verified through an animal infection treatment experiment. The Chinese herbal medicine and mixture I screened against Bacillus paracarinis could be used as feed additives to protect against the occurrence of infectious rhinitis in chicken farms, and provide a reference for further research and development of new veterinary drugs to replace antibiotics for the treatment of infectious rhinitis in chickens.

## Conclusions

This study successfully isolated a strain of Avibacterium paragallinarum from diseased chickens. Through drug sensitivity tests, the strongest antibacterial effect of the traditional Chinese medicine mixture I was selected, and the preventive and therapeutic effects of the traditional Chinese medicine mixture I were verified through animal infection treatment experiments.

## CRediT authorship contribution statement

**Zipeng Yue:** Writing – original draft, Resources, Investigation, Data curation. **Yurong Li:** Writing – review & editing. **Ligong Chen:** Writing – review & editing. **Tian Liang:** Resources. **Yinghui He:** Formal analysis. **Linyao Hao:** Resources. **Ying Jiang:** Resources. **Tianyang Li:** Resources. **Shuying Huo:** Writing – review & editing, Supervision, Formal analysis.

## Disclosures

All authors have accepted responsibility for the entire content of this manuscript and approved its submission. The authors declare that they have no known competing financial interests or personal relationships that could have appeared to influence the work reported in this paper.
